# Metabolic benefits of methionine restriction in adult mice do not require functional methionine sulfoxide reductase A (MsrA)

**DOI:** 10.1038/s41598-022-08978-4

**Published:** 2022-03-24

**Authors:** Kevin M. Thyne, Adam B. Salmon

**Affiliations:** 1 Sam and Ann Barshop Institute for Longevity and Aging Studies, University of Texas Health San Antonio, San Antonio, TX 78229 USA; 2 Department of Molecular Medicine, University of Texas Health San Antonio, San Antonio, TX 78229 USA; 3 Geriatric Research Education and Clinical Center, Audie L. Murphy Hospital, South Texas Veterans Health Care System, San Antonio, TX 78229 USA

**Keywords:** Molecular medicine, Chemical modification, Ageing, Metabolism, Homeostasis

## Abstract

Methionine restriction (MR) extends lifespan and improves several markers of health in rodents. However, the proximate mechanisms of MR on these physiological benefits have not been fully elucidated. The essential amino acid methionine plays numerous biological roles and limiting its availability in the diet directly modulates methionine metabolism. There is growing evidence that redox regulation of methionine has regulatory control on some aspects of cellular function but interactions with MR remain largely unexplored. We tested the functional role of the ubiquitously expressed methionine repair enzyme methionine sulfoxide reductase A (MsrA) on the metabolic benefits of MR in mice. MsrA catalytically reduces both free and protein-bound oxidized methionine, thus playing a key role in its redox state. We tested the extent to which MsrA is required for metabolic effects of MR in adult mice using mice lacking MsrA. As expected, MR in control mice reduced body weight, altered body composition, and improved glucose metabolism. Interestingly, lack of MsrA did not impair the metabolic effects of MR on these outcomes. Moreover, females had blunted MR responses regardless of MsrA status compared to males. Overall, our data suggests that MsrA is not required for the metabolic benefits of MR in adult mice.

## Introduction

Restriction of dietary intake of the amino acid methionine, even in the absence of restriction of calories, has been shown to consistently extend lifespan and improve metabolic health in rodents. Previous studies have shown that dietary methionine restriction (MR) in rodents can extend lifespan up to 42% compared to rodents fed diets replete with methionine^1–3^. Moreover, MR has strong effects on metabolic function and has been shown to improve glucose homeostasis^4–9^, decrease oxidative stress^2,10–15^, and promote adipose tissue browning^16–22^. These findings are consistent with the idea that dietary intervention by restricting methionine improves health in addition to extending longevity. Understanding the molecular mechanisms responsible for the physiological effects of MR could then have significant impact as potential targets to improve human health throughout life.

While pro-longevity dietary interventions affect many molecular pathways consistent with the pillars of aging, it seems likely that the effects of MR may strongly affect methionine-dependent pathways. Methionine metabolism has multiple roles in regulating physiological function. Methionine is the initiating amino acid in protein translation, and restriction of this amino acid in the diet has been shown to reduce protein synthesis^23–27^. MR has also been shown to increase activity of pathways involved in protein degradation and recycling including the ubiquitin proteasome system and autophagy consistent with enhanced proteostasis^28^. Methionine plays a significant role in the generation of hydrogen sulfide via conversion to cysteine through the transsulfuration pathway. Hydrogen sulfide and its generation has been shown to play a central role in the effects of dietary restriction including calorie restriction (CR) and MR^29^. Methionine is also used in the generation of *S*-adenosyl methionine (SAM) which is the primary methyl group donor for methyltransferases. The effects of MR on this metabolite are complex with levels being decreased in some cases^30–32^ while having no impact in others^31,33^, and with MR either increasing global DNA methylation^30^ or having no effect^12,30^. There is also evidence that other interventions that extend lifespan significantly alter methionine metabolism. For example, Ames dwarf mice, which live more than 40% longer than their controls due to a mutation in *Prop1*, have also been shown to have altered sulfur metabolism and higher SAM turnover^34,35^. Interestingly, Ames dwarf mice show no impact of MR on glucose metabolism^36^ or longevity^34^ suggesting perhaps overlap in pro-longevity mechanisms of these interventions.

The potential role of methionine reduction–oxidation (redox) regulation in the mechanism of MR has been relatively unexplored. Methionine itself is extremely susceptible to oxidation due to the sulfur atom that makes up its amino acid side chain. Under oxidizing conditions, the pro-chiral sulfur of methionine’s thioether can be oxidized to yield two epimers of L-methionine sulfoxide^37^. Methionine sulfoxide reductases (Msr) have evolved to catalytically reduce methionine sulfoxide to methionine. This is an important oxidative damage repair mechanism as methionine and methionine sulfoxide differ in structure and polarity. In addition, there is growing evidence that redox regulation of methionine residues may also act as functional regulators of proteins^38–42^. At least four forms of Msr have evolved in mammals: MsrA which reduces the chiral (S) epimer of methionine sulfoxide and MsrB1-3 which reduce the (R) epimer. Of these, MsrA is the most ubiquitously distributed among mammalian tissues and the most well-studied Msr. The lack of MsrA does increase sensitivity to oxidative challenge and results in increased oxidation of methionine residues^43^. Interestingly, MsrA has also been reported to play a role in the regulation of glucose and insulin signaling under metabolic challenge. The lack of MsrA in mice exacerbates insulin resistance caused by high fat feeding in part by failing to prevent oxidative damage to key insulin signaling proteins^44^. Conversely, overexpression of MsrA preserves insulin sensitivity in mice fed a high fat diet^45^.

Relatively little is known regarding the potential role of methionine redox metabolism or Msr activity in the outcomes of MR. MR in mice reduces plasma methionine and methionine sulfoxide levels to approximately the same degree^31^. It has also been shown that the lack of both MsrA and MsrB1, but not either independently, can hinder growth rate of weaned mice under MR^33^, suggesting an important role of Msr enzymes in preserving methionine during restriction. Here, we directly tested the functional requirement for MsrA on the metabolic outcomes of MR in mice. Using mice lacking MsrA expression (*MsrA* KO), we find that MsrA is not required to reduce body weight or composition under MR. Moreover *MsrA* KO mice responded as well, or better, to MR in terms of improvement in glucose metabolism and respiration. Together, our studies suggest that functional MsrA is not required for the metabolic benefits of MR.

## Results

**Figure 1:**
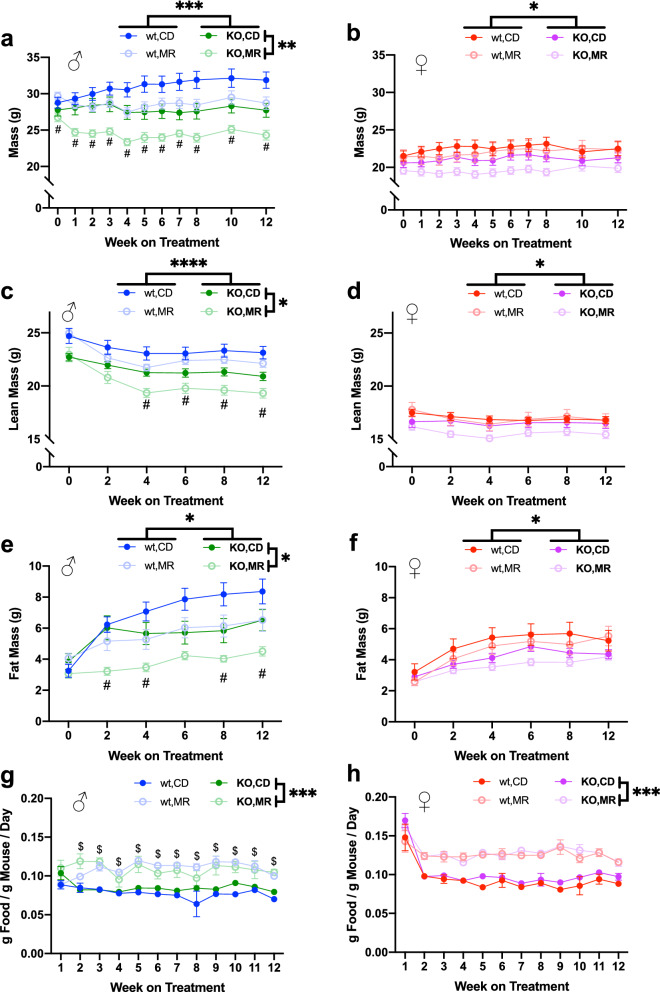
MR has sex-specific effects on physiological measures. Body weight (**a**), (**b**) and normalized food consumption (**g**), (**h**) measured weekly while on MR diet. Body composition data for fat mass (**c**), (**d**), lean mass (**e**), (**f**) measured every two weeks. Sexes analyzed separately with Repeated Measures, 3-Way ANOVA. Main effects for diet and MsrA KO genotype indicated by significance bars by legends (right side and above, respectively). Post-hoc diet specific effect within a genotype was analyzed with Repeated Measures, 2-Way ANOVA Multiple Comparisons with False Discovery Rate correction, Q = 0.05. Means at specific time points being significantly different (*p* < 0.05) between diets for wild type denoted by dollar sign ($) and for MsrA KO denoted by a pound sign (#). Graphs represent means ± s.e.m, and all groups were 8–10 mice. (*p < 0.05; **p < 0.01; ***p < 0.001; ****p < 0.0001).

In this study, we used adult mice to potentially differentiate the effects of MR on metabolic function from those of MR-mediated delays in development^2^. At an average 7.5 months of age, fully grown mice began dietary interventions of either MR (0.15% methionine as proportion of protein) or composition equivalent control diet (CD) which was replete with levels of methionine (0.86% methionine as proportion of protein) approximately equivalent to that found in normal chow. During the study, diets were provided ad libitum and food consumption was monitored throughout. Prior to intervention with each diet, both male and female *MsrA* KO mice were lower in body weight than their wild type counterparts (Fig. 1a, b). In wild type males, MR reduced body weight and fat mass compared to CD, consistent with previous reports^1,5,6,17,18,21,22,30^. In addition, MR caused a small change in lean mass compared to CD. Surprisingly, MR induced reduction of male body weight, fat mass, and lean mass in *MsrA* KO to a greater extent than in wild type mice—the percentage change in these outcomes was larger in *MsrA* KO males compared to wild type males (Fig. 1a, c, e). MR did not result in significant changes body weight, fat mass, or lean mass regardless of genotype during the 3 months of treatment in females (Fig. 1d, f).

Despite the reported body weight reduction in the MR groups, we also found that MR increased food consumption in all MR groups compared to their sex- or genotype-equivalent CD groups (Fig. 1g, h). This increase in food consumption when normalized to body weight was stable throughout the three months the mice were on the diet. While MR significantly increased food consumption compared to CD, post-hoc analysis of these data indicated that only the wild type males had a significant difference between the MR and CD. These results are similar to others reported in regards to body composition and food consumption under MR^1,5,6,17,18,21,22,24,30^.

**Figure 2:**
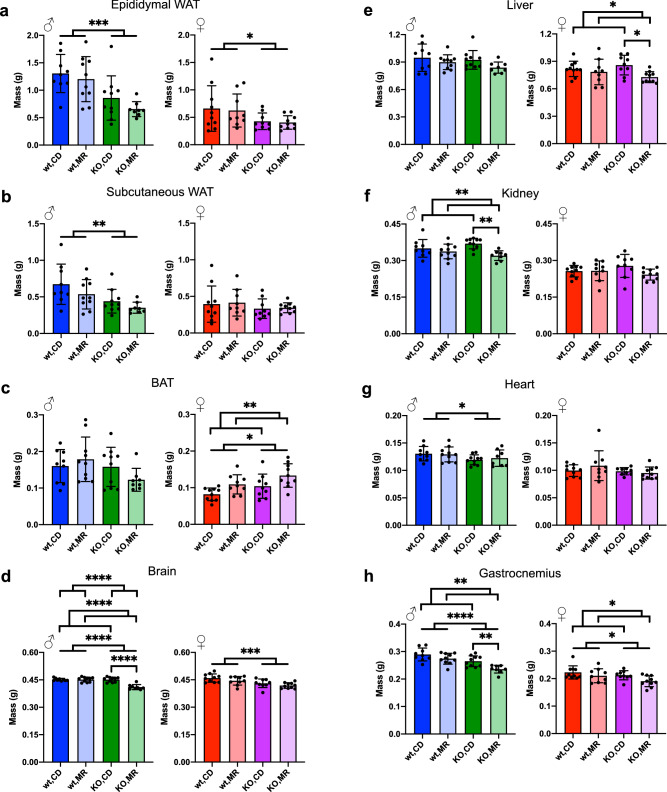
MR has sex-specific effects on tissue masses. Tissue masses for Epididymal WAT (**a**), Subcutaneous WAT (**b**), BAT (**c**), Brain (**d**), Liver (**e**), Kidney (**f**), Heart (**g**), and Gastrocnemius (**h**) were measured at time of collection. Analysis was within each sex via Two-Way ANOVA for main effects. Post-hoc analysis was performed with Sidac multiple comparisons correction to assess the diet effect within each genotype. Graphs represent means ± s.d. and all groups were 8–10 mice. (*p < 0.05; **p < 0.01; ***p < 0.001; ****p < 0.0001).

The post-mortem tissue masses reflected the decreased fat mass observed with QMR (Fig. 1e, f) with epididymal and subcutaneous white adipose tissue (WAT) being lower for the *MsrA* KO males, but with only a small difference observed in the *MsrA* KO female subcutaneous WAT (Fig. 2a, b). Brown adipose tissue (BAT) was significantly increased in females by MR (Fig. 2c) but not males; previous studies have been equivocal in reporting this the response of BAT to MR^4,16,46^. Surprisingly, MR resulted in a dramatic decrease in brain mass for the male MsrA KO mice, but the reason for this is unclear (Fig. 2d). MR also resulted in sex-specific decreases in liver and kidney mass—a decrease in liver mass in the females and a decrease in kidney mass in the males (Fig. 2e, f). The change in heart mass and gastrocnemius mass (Fig. 2g, h) was consistent with the lean mass decrease observed with QMR (Fig. 1c, d).

**Figure 3:**
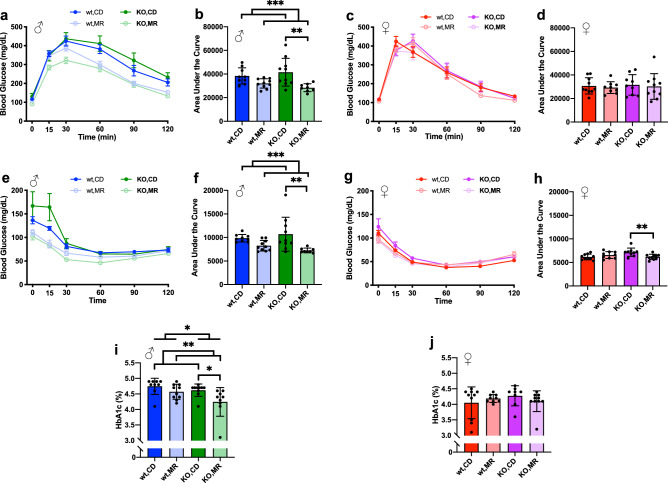
Males have greater glucose metabolism response to MR than females. Glucose Tolerance Test curves (**a**), (**c**) and Area Under the Curve (AUC) for their respective curves (**b**), (**d**) for both sexes. Insulin Tolerance Test curves (**e**), (**g**) and AUC for their respective curves (**f**), (**h**). AUC calculated by the trapezoid method and analyzed within each sex via Two-Way ANOVA for main effects. Post-hoc analysis was performed with Sidac multiple comparisons correction to assess the diet effect within each genotype. HbA1c was analyzed similarly (**i**), (**j**). Line graphs represent means ± s.e.m., bar graphs represent means ± s.d.. Groups were 8–10 mice. (*p < 0.05; **p < 0.01; ***p < 0.001).

Similar to previously reports, glucose metabolism was improved by MR even when started in non-obese adult animals^4–6,9,22^. In males, glucose tolerance tested (GTT) after 9 weeks of intervention was improved when MR groups were analyzed as a whole (Fig. 3a, b). Post-hoc analysis of the within-sex, two-way ANOVA showed a significant effect of MR in *MsrA* KO (*p* = 0.0047) though not in wild type mice (*p* = 0.1545) (Fig. 3b). MR also improved insulin sensitivity in males as measured by insulin tolerance test (ITT) following 11 weeks of dietary intervention (Fig. 3e, f). Similar to GTT results, there was a significant post-hoc effect of MR on *MsrA* KO mice (*p* = 0.0012) though not in wild type mice (Fig. 3f). In contrast to results in males, MR had no significant main effect on GTT or ITT in female mice for either wild type or *MsrA* KO mice (Fig. 3c, d, g, h). To assess for potential differences in the molecular pathways regulating insulin sensitivity, we also measured phosphorylation of Akt. Western blots were performed to assess Akt phosphorylation, and we found no effect of MsrA on possible molecular mechanisms of insulin sensitivity. Phosphorylation of Akt in fasted animals was unaffected by either diet or genotype in males; in females, phosphorylation was increased in *MsrA* KO compared to wild type (Supplemental Fig. S4a). In skeletal muscle, we found no significant change in either males or females, in line with our glucose metabolism results (Supplemental Fig. S5a). We also measured glycated hemoglobin A1c (HbA1c) as an additional marker of glucose metabolic function, and found HbA1c significantly lower in male mice on MR compared to CD (Fig. 3i) but there was no effect of diet in females (Fig. 3j). Interestingly, *MsrA* KO males had lower HbA1c than their wild type male counterparts overall, although there was no difference between *MsrA* KO and wild type females.

**Figure 4:**
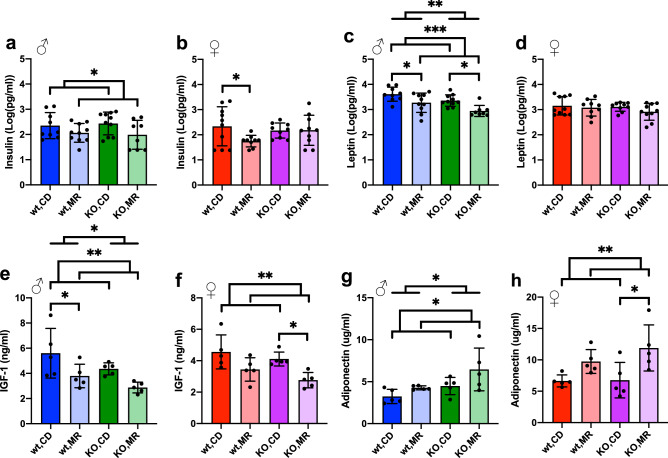
MR alters metabolic markers more in males. MilliPlex of serum from mice overnight fasted at time of sacrifice. Selected panel results of Insulin (**a**), (**b**) and Leptin (**c**), (**d**) measured by MilliPlex. Data was log transformed before analysis to preserve normality. Data points at lower end of detection were included as lowest value given by the assay’s internal standard curve. IGF-1 (**e**), (**f**) and Adiponectin (**g**), (**h**) measured in plasma by ELISA. Analysis was within each sex via Two-Way ANOVA for main effects. Post-hoc analysis was performed with Sidac multiple comparisons correction to assess the diet effect within each genotype. Graphs represent means ± s.d. and all groups were 8–10 mice for MilliPlex, and 5 mice per group for IGF-1 ELISA. (*p < 0.05; **p < 0.01; ***p < 0.001).

After 12 weeks of MR, plasma was collected after overnight fast to assess endocrinological effects of MR in wild type and *MsrA* KO mice. MR significantly reduced insulin concentrations in males compared to CD (Fig. 4a). In females, MR reduced insulin concentration in wild type females but not *MsrA* KO (Fig. 4b). These results are generally in line with the idea that MR improves glucose metabolic function *in vivo*^4–6,9,22^. Leptin, a peptide secreted from adipose tissue and involved in satiety, was lower in the MR males compared to CD males, but unchanged by diet in females (Fig. 4c, d). Serum concentrations of IGF-1 were decreased with MR in both sexes as has been shown in other studies^2,8,46–49^ and is associated with increased lifespan^34,49^, however there was only a significant genotype effect of the *MsrA* KO in the males (Fig. 4e, f). Adiponectin was increased with MR in both sexes as has been shown in other studies^8,18,46^, with a genotype effect observed in the males with the *MsrA* KO having higher concentrations of adiponectin compared to wild type (Fig. 4g, h). The effect of MR on other endocrine factors associate with metabolic function were largely unchanged in with diet or genotype in male mice. However, MR did affect these outcomes in female mice with both genotype and diet dependent effects (Supplemental Fig. S1).

MR has been shown to decrease oxidative stress in numerous studies ^2,10–15^. Given the role of MsrA in repairing/regulating oxidative damage to methionine, we tested whether the lack of MsrA altered the effect of MR on such outcomes (Supplemental Figs. S4, S5). We found no effect of MR or lack of MsrA on the content of GPX1 or SOD2 expression in liver (Supplemental Fig. S4b, e). In line with reducing oxidative stress, we found MR decreased GPX4 expression in males, but not females (Supplemental Fig. S4c). As a measure of oxidative damage, we measured 4-hydoxynonal (HNE; Supplemental Fig. S4d) adducts, the result of lipid oxidation. We found an interaction effect detected in males between diet and genotype though no effect of either alone. This suggests some possible benefit of *MsrA* KO, but more studies would be required to determine this. In muscle, we found no effect of diet or genotype on HNE (Supplemental Fig. S5b), but did indicate that *MsrA* KO had decreased SOD2 expression in males only (Supplemental Fig. S5c). Together these results suggest the main effects reported are not due to dramatic changes in oxidative stress or damage.

**Figure 5:**
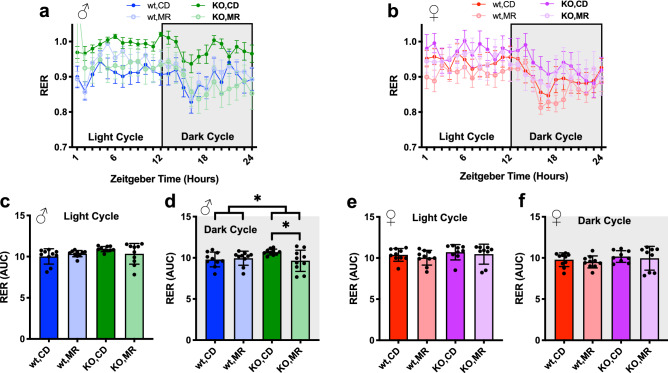
MR had minimal impact on RER. Respiratory Exchange Ratio (RER) for 24 h period (**a**), (**b**), analyzed via 3-Way ANOVA for light and dark cycle independently. Graphs represent mean ± s.e.m. Male light cycle (**c**) and dark cycle (**d**) AUC, and Female light cycle (**e**) and dark cycle (**f**) analyzed within each sex via Two-Way ANOVA for main effects on Area Under the Curve (AUC) for RER. Post-hoc analysis was performed with Sidac multiple comparisons correction to assess the diet effect within each genotype. Line graphs represent means ± s.e.m., bar graphs represent means ± s.d., and all groups were 9–10 mice on diet for ~ 8 months. (*p < 0.05).

**Figure 6:**
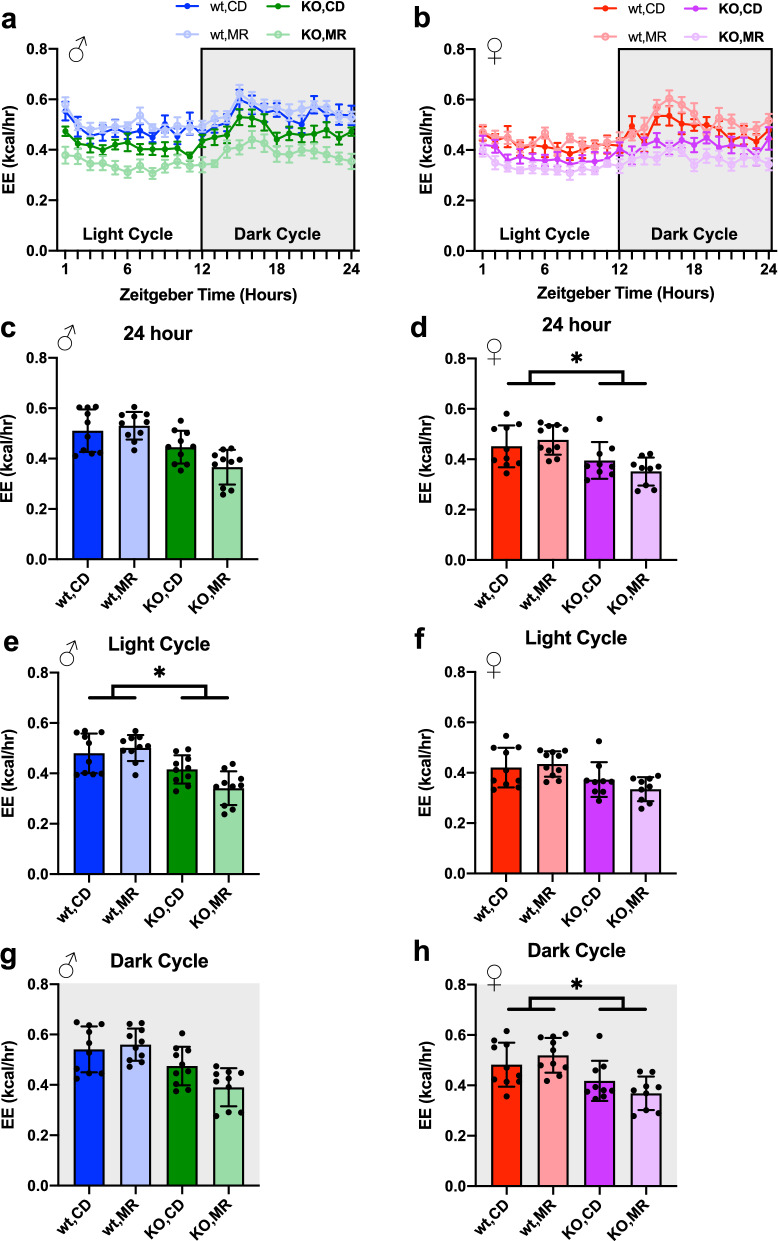
Energy Expenditure was not impacted by MR. Energy Expenditure (EE) for 24 h period based on VO2 not normalized to mass for mice from Cohort 2 (**a**), (**b**). Data averaged over the 24 h test period (**c**), (**d**), and for the light (**e**), (**f**) and dark (**g**), (**h**) cycle separately. Analyzed with ANCOVA using lean mass as a control variable for AUC graphs of EE for full 24 h as well as light and dark cycles. Line graph represents mean ± s.e.m., bar graphs represent mean ± s.d. Groups were 9–10 mice. (*p < 0.05).

We also addressed the effect of MR and lack of MsrA on whole animal metabolism as an additional assessment of metabolic function. Using an independent cohort of mice treated identically, we measured respiration of animals following 8 months of MR. Overall, MR did not affect respiratory exchange ratio (RER) compared to CD (Fig. 5) with the exception of a decrease in dark cycle RER in MsrA KO males on MR (Fig. 5d). Oxygen consumption (VO2) and carbon dioxide production (VCO2) were also analyzed normalized to body weight (Supplemental Fig. S2) and lean mass (Supplemental Fig. S3), with only lean mass normalization showing significant effect of genotype but not diet. Energy expenditure (EE) was calculated from this data and averaged over 24 h, as well as broken into the light and dark cycle averages (Fig. 6). ANCOVA analysis controlling for lean body mass yielded a main effect of the MsrA KO for light cycle EE (Fig. 6e), but had a nearly significant genotype effect for overall 24 h average EE. Females had a similar outcome with a main effect of MsrA KO for the dark cycle and overall 24 h average EE (Fig. 6d, h). Taken together these results indicate that MR did not significantly alter EE, while MsrA KO had a more profound effect when controlled for lean body mass (Fig. 6).

## Discussion

Insight regarding the molecular underpinnings of MR have the potential to significantly advance our understanding of the physiological benefits of this intervention including those on longevity and metabolic function. Here we tested whether the methionine sulfoxide reducing enzyme MsrA impacts the effect of MR on metabolism in mice. Overall, we found that mice lacking MsrA have normal, or potentially even greater, response to MR compared to wild type mice in terms of weight loss, glucose and insulin response, and respiration. One interpretation of these outcomes could be that MsrA is dispensable for the effects of MR on glucose metabolism in vivo, or that the impact of MsrA modulation by MR is on functions that do not directly affect glucose metabolism. In regards to the potential lack of requirement of this enzyme, Zhao et al*.* showed previously that lack of MsrA did not significantly impact growth from weaning under methionine restricted conditions, a classical bioassay for growth^33^. This report showed also that the lack of MsrB1 alone had no effect in this assay, but that the lack of both MsrA and MsrB1 delayed growth under methionine restriction from weaning. Together, these data suggest a functional requirement for Msr in general under conditions of limiting methionine, and also that there may be compensatory mechanisms for this process among Msr enzymes. Data from MsrB1 KO mice showed that other Msr enzymes are upregulated in a tissue specific manner^50^. More work in this area would be of benefit to understanding the role of Msr and methionine metabolism in general in MR.

Among potential mechanisms by which MR alters physiological function are reported improvements in resistance and response to oxidative stress induced by MR^2,10,11,15,43^. While not a primary antioxidant, MsrA has been shown to play an important role in the resistance to oxidative stress and the lack of MsrA reduces resistance to oxidant challenge in mice^43,44^. Studies have shown that loss of any of these Msr enzymes under normal diet conditions is survivable, but can make the organism more sensitive to oxidative stress. Counterintuitively, knockout of the four Msr enzymes, MsrA and MsrB1-3, has been shown to increase oxidative stress resistance, presumably through compensatory mechanisms among the antioxidant defense system^51^. In addition to repairing oxidized methionine, Msr have been proposed to act more generally in oxidant defense as part of a methionine redox system^52^. In this scenario, methionine acts as a “free-radical sink” taking on oxidative damage to protect other macromolecules in the cell. These methionine residues can then be recycled (reduced) by Msr to further defend the cell from oxidant damage. Under this paradigm, it is conceivable that restriction of methionine then increases the importance of functional Msr to prevent oxidative damage. While we did not address this here, it would be of interest to assess the interplay of MR and MsrA under such oxidant challenges.

While our results in general suggest MsrA is not required for the effects of MR on metabolism, some of our results do suggest potential roles in mediating other functions affected by MR. For example, MR in wild type mice did not significantly affect lean mass throughout our study. On the other hand, *MsrA* KO mice lost a significantly quantity of lean mass under MR that did not return to normal levels through the duration of study (Fig. 1c). Generally, loss of lean mass might be associated with declines in muscle mass, function, etc. We did observe a decrease in gastrocnemius mass at time of tissue collection in both sexes with MR which was significant in MsrA KO post-hoc testing (Fig. 2h). A similar decrease in mass has been reported in other MR studies^4,7,47^. Further examination of muscle function and structure will be necessary to delineate this potential novel relationship between methionine metabolism and muscle. In particular, assessment of late-life muscle function and the effect of MR with Rota-Rod and grip strength would expand on our understanding of whether this intervention improves overall healthy aging. Fat mass was also shown to decreased by MR (Fig. 1e, f) as measured by QMR. However, the individual tissue masses did not corroborate this (Fig. 2a–c). This could have been the result of how the tissue was collected as well as that the QMR fat signal measured the entire organism. Contributions from other tissues or fat depots not collected could have resulted in this discrepancy. In addition, it will be of interest to determine whether the MR-mediated declines in kidney, liver, and brain mass of *MsrA* KO mice have significant impact on the function of these organs in vivo.

MR has been shown to mediate many of its functional benefits through modulation of endocrine regulation. For example, MR has been shown to increase circulating concentrations of adiponectin^8,18,46^; this adipokine then can directly improve glucose metabolism. MR has also been shown to reduce IGF1^2,8,46,47^, likely through down-regulation of growth hormone signaling pathways. While IGF1 is more directly associated with improved glucose metabolism, the relationship between low IGF1 and improved glucose metabolism shown with MR likely reflects reduced concentrations of the pro-diabetogenic growth hormone. More generally, our results show a varied, and sex-dependent, response to MR in terms of most endocrine outcomes measured in our comprehensive endocrinological assays.

In this study, the response to MR on physical and functional outcomes was more pronounced in male mice compared to female mice regardless of genotype. Some evidence of this can be seen in a recent study by Forney et al*.* which also tested the response to MR in both sexes^17^. Similar to our results, this previous study showed that MR has significantly different functional impact on each sex and in general that males respond more robustly to MR than do females. This suggests a significant interaction between these factors and the animal’s sex, but it remains an open question if this is driven by sex hormones or sex chromosomes. The lack of MsrA had no effect on these sex-differences, suggesting that this enzyme does not affect the central mediators of this MR and genetic sex relationship. Further experiments would be required to better understand the interactions of methionine sulfoxide reductases and sex with the effects of MR. Some possible areas of interest could be gleaned from studies on how MsrA mutations impact human health. We have previously shown that MsrA plays a role in the metabolic response to diet-induced obesity in mice^44,45^. Decreases in MsrA have been associated with pathologies of the eye lens and have been implicated in Alzheimer’s Disease^53–55^. GWAS have also implicated MsrA mutants in rheumatoid arthritis^56^. These studies provide possible areas of investigation to better understand the interactions with MR.

Overall, our results indicate that (1) MsrA is not functionally required for the effects of MR on metabolic function and its loss may enhance certain effects, (2) MR can significantly improve body composition and metabolic function even when administered to normal adult rodents, (3) in general, males respond to a greater degree to MR than do females in terms of metabolic function. While our studies investigated these interactions and effects in adult mice, it remains an open question as to their long-term effects on longevity and health span. While previous studies have indicated that lack of MsrA does not negatively impact lifespan, its interaction with MR is uncertain.

## Methods

### Animal usage and ethical procedures

All animal experiments were approved by the Institutional Animal Care and Use Committees and UTHSA (Animal Protocol 20170190AR), and have been reported following ARRIVE guidelines. All methods were conducted in accordance with international ethical standards and guidelines.

### Animal experiments

#### Cohort 1

Genetic mutant mice with homozygous deletion of MsrA were maintained on C57Bl/6 J background as previously reported^44^. For this study, both male and female mice were used, with wild-type C57Bl/6 J as controls. All mice were confirmed for genotype by PCR analysis of tail-derived genomic DNA. Animal studies were performed in a specific pathogen-free vivarium maintained at 25 °C with a 12 h:12 h light:dark cycle. Mice were maintained in ventilator cages at density of 3–4 (male) or 5 (female) mice per cage, and provided food and water ad libitum throughout study except prior to metabolic assessment. Mice were maintained on standard chow (NIA-31 equivalent) until an age of approximately 7.5 months post-weaning until cages were assigned to either control diet (CD) (0.86% Met, TestDiet 578F w/0.86% MET—5SFD) or MR (0.15% Met, 0% Cys, Test Diet 96D2, modified TestDiet 58B0) diet for the duration of the study. 9–10 mice were assigned to each group combination of sex, genotype, and diet, resulting in 8 groups of 9–10 mice. Cages were assigned non-randomly to have approximately equal starting weights between diet groups within each genotype. Body weight and composition (by quantitative magnetic resonance, Echo MRI, Houston TX) were performed prior to initiation of dietary intervention. During the study, mice were weighed weekly, food consumption was measured weekly, and body composition was measured bi-weekly. Tissues were collected after euthanasia via CO2 asphyxiation, and measured for mass before being snap frozen in liquid nitrogen for storage. During the course of the study one male mouse suffered from rectal prolapse and subsequent weight loss. Data collected from this mouse was censored from the study.

#### Cohort 2

An identical cohort of mice was started at the same time using the same experimental paradigm. These mice were used for measurement of oxygen consumption after being treated with MR or CD for approximately 8 months. Body weight and food consumption were measured in these mice on the same schedule as the first group. Body composition was measured monthly.

#### Glucose tolerance (GTT) and insulin tolerance tests (ITT)

Following an overnight (16 h) fast, fasting blood glucose concentrations were measured by tail bleed using an AimStrip Plus digital glucose meter. For GTT mice injected intraperitoneally with 1.5 mg/g body weight glucose in PBS. Blood glucose was measured by tail bleed at 15, 30, 60, 90, and 120 min post injection. Area Under the Curve (AUC) was calculated via the trapezoid method. ITT were performed similarly with intraperitoneal injection of 0.75U Insulin/g body weight in PBS and measurement of blood glucose by tail bleed following 15, 30, 60, 90, and 120 min. GTT were performed two weeks prior to ITT in the same animals.

#### Hemoglobin A1c

At the completion of study, mice were fasted overnight and tail bleed was performed to collect whole blood in EDTA washed tubes and temporarily stored on ice. Collected blood was allowed to warm to room temperature before HbA1c was measured on a Siemens Vantage DCA Analyzer (Siemens AG, Munich, Germany).

#### MilliPlex metabolic panel

At the completion of study, mice were fasted overnight. Blood was collected and centrifuged in EDTA-coated tubes then flash frozen. Plasma was then tested in the MilliPlex MMHMAG-44 K MILLIPLEX MAP Mouse Metabolic Hormone Panel (EMD Millipore). Data was log transformed to preserve normality prior to analysis.

#### ELISA

IGF1 ELISA (Abcam, ab100695) and Adiponectin ELISA (Abcam, ab108785) were performed as per manufacturer’s instructions.

#### Oxygen consumption

Oxygen consumption was performed on mice after eight months on diet. Oxygen consumption and carbon dioxide generation was measured via the CLAMS system (Columbus Instruments). Mice were individually housed during testing and were housed for 24 h before measurement was started. Oxygen and carbon dioxide volume were measured for the next 24 h after acclimatization. Measurements were based on 1 h bins.

#### Energy expenditure

Energy expenditure was calculated based on lean mass from QMR and VO2 following equations provided by the CLAMS system. Briefly, EE was calculated based as 3.815 + 1.232 * RER * VO2, with VO2 not normalized to mass.

#### Western blot

Samples were prepared by homogenizing ~ 50 mg of tissue in ~ 550ul (11ul:1 mg) RIPA for skeletal muscle and ~ 500ul (10ug:1 mg) RIPA for liver. RIPA contained protease and phosphatase inhibitor cocktail (Pierce). Tissue samples homogenized using a TissueLyserII (Qiagen) for 2 min at 30 Hz for skeletal muscle and 1 min at 30 Hz for liver. Supernatant after centrifugation was collected and protein measured using Pierce BCA assay (Bio-Rad).

Blotting was performed with Criterion TGX gels (Bio-Rad). Gels were transferred to PVDF membranes (Bio-Rad) and total protein measured with Ponceau S (Sigma) staining imaged with a Perfection V39 flatbed scanner (Epson). Membranes were blocked with 10% non-fat dry milk or 2% BSA in TBST. Membranes were incubated with primary antibody overnight at 4 °C with agitation: Akt (Cell Signaling, 9272), SOD2 (AbCam, ab13533), GPX4 (Santa Cruz Biotechnology, sc-27529), pAkt(S473) (Cell Signaing, 9271), GPX1 (AbCam, ab22604), HNE (Alpha Diagnostic, HNE11-S). Membranes were washed with TBST and incubated with HRP secondary (Santa Cruz Biotechnology) for 1 h at room temperature. Membranes were then washed and developed with Pierce ECL Plus Western Blotting Substrate (ThermoFisher). Membranes were imaged on a Typhoon FLA 7000 (Amersham). All quantifications were performed in ImageStudioLite (LI-Cor).

#### Statistics

Statistics were completed using Prism 8. Physiological measures of body weight/composition and food consumption were measured within sex via a Repeated Measures, 3-Way ANOVA with post-hoc multiple comparisons performed with the False Discovery Rate set Q = 0.05. All other tests were performed using 2-Way ANOVA within each sex with post hoc tests to assess diet effect within genotype, Sidac corrected. Energy Expenditure was analyzed with the “car” package in R to perform an ANCOVA controlling for lean body weight as a covariate.

## Supplementary Information

Supplementary Information.

## Data Availability

The data presented in the work are available from the corresponding author upon request.
