# Supplementary material for: Metabolic benefits of methionine restriction in adult mice do not require functional methionine sulfoxide reductase A (MsrA)
Source: Sci Rep. 2022 Mar 24;12:5073. doi: 10.1038/s41598-022-08978-4 (PMC8948287; doi:10.1038/s41598-022-08978-4)
Supplement: Supplementary file 1 — Supplementary Information. [file 41598_2022_8978_MOESM1_ESM.pdf]

Supplementary Information

Metabolic benefits of methionine restriction in adult mice do not require functional methionine sulfoxide reductase A (MsrA)

Kevin M. Thyne [1], Adam B. Salmon [1,2,3,*]

[1] Sam and Ann Barshop Institute for Longevity and Aging Studies, University of Texas Health San Antonio, San Antonio, Texas, 78229, USA
[2] Department of Molecular Medicine, University of Texas Health San Antonio, San Antonio, Texas. 78229, USA
[3] Geriatric Research Education and Clinical Center, Audie L. Murphy Hospital, South Texas Veterans Health Care System, San Antonio, Texas, 78229, USA

*Corresponding author

Kevin Thyne – Thyne@livemail.uthscsa.edu
*Dr. Adam Salmon – Salmona@uthscsa.edu

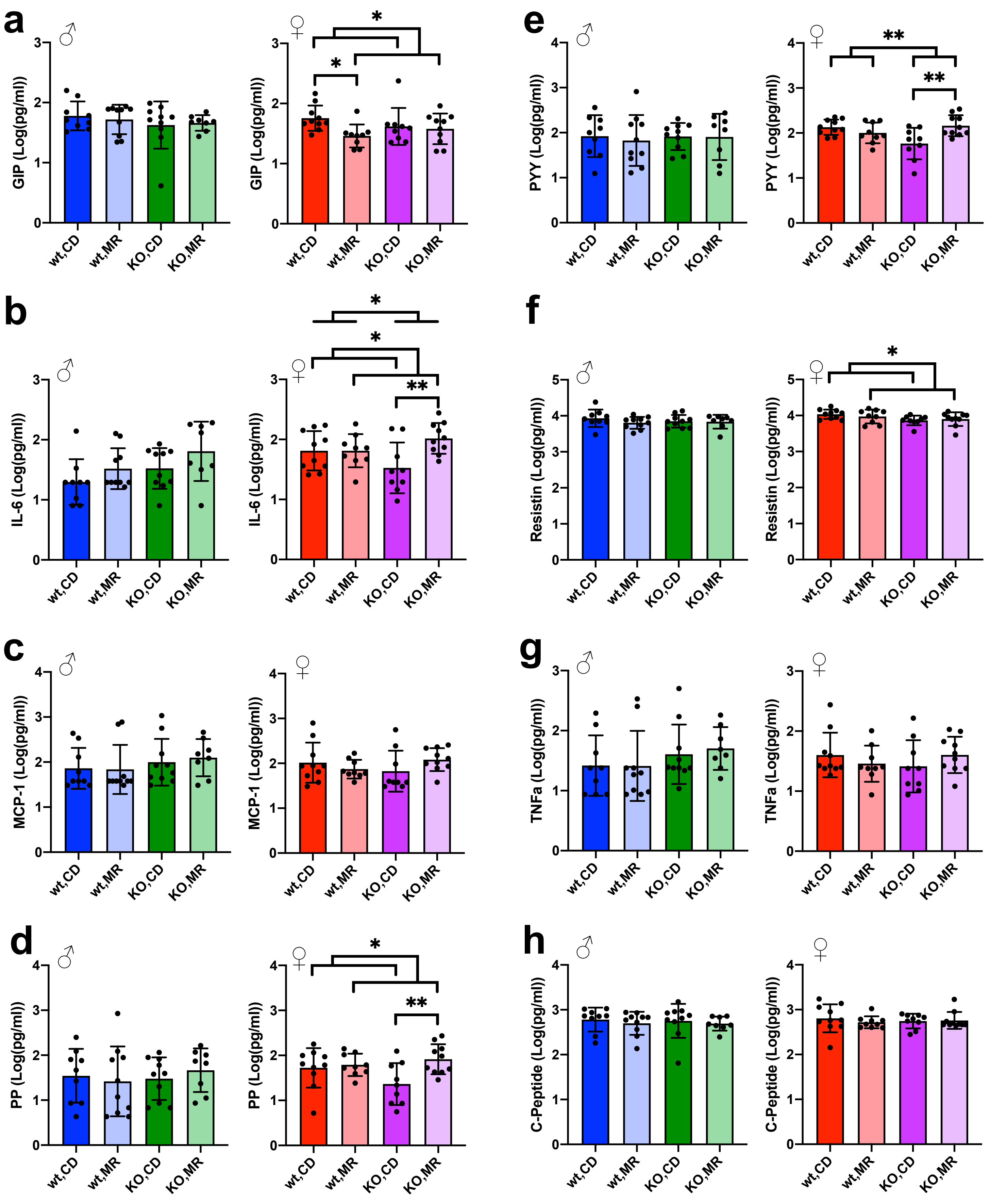

Supplemental Figure S1 – MR and MsrA altered metabolic and inflammation markers in a sex-specific manner

MilliPlex of serum from mice overnight fasted at time of sacrifice. Selected panel results of GIP (**a**), IL-6 (**b**), MCP1 (**c**), PP (**d**), PYY (**e**), Resistin (**f**), TNFα (**g**), and C-Peptide (**h**) measured by MilliPlex. Data was log transformed before analysis to preserve normality. Data points at lower end of detection were included as lowest value given by the assay's internal standard curve. Analysis was within each sex via Two-Way ANOVA for main effects. Post-hoc analysis was performed with Sidac multiple comparisons correction to assess the diet effect within each genotype. Graphs represent means ± s.d. and all groups were 8-10 mice. (*p < 0.05; **p < 0.01)

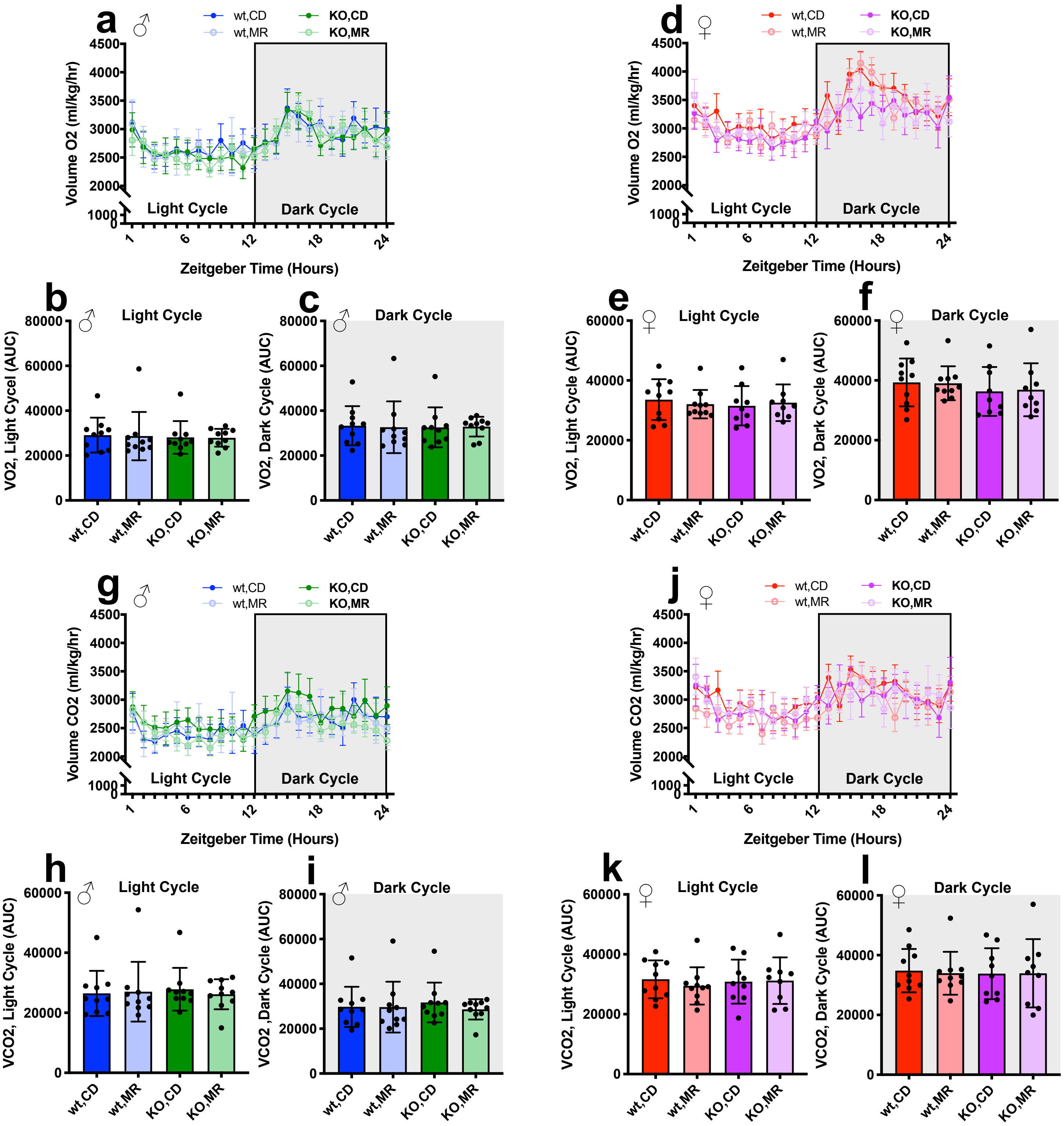

Supplemental Figure S2 – MR and MsrA did not alter body weight normalized respirometry
Oxygen consumption (VO2) and carbon dioxide production (VCO2) for 24hr period normalized to body weight, analyzed via 3-Way ANOVA for light and dark cycle independently (**a**,**d**,**g**,**j**). Graphs represent mean ± s.e.m. Male and female light cycle and dark cycle Area Under the Curve (AUC) were analyzed within each sex via Two-Way ANOVA for main effects on AUC for VO2 (**b**,**c**,**e**,**f**) and VCO2 (**h**,**i**,**k**,**l**). Post-hoc analysis was performed with Sidac multiple comparisons correction to assess the diet effect within each genotype. Line graphs represent means ± s.e.m., bar graphs represent means ± s.d., and all groups were 9-10 mice on diet for ~8 months.

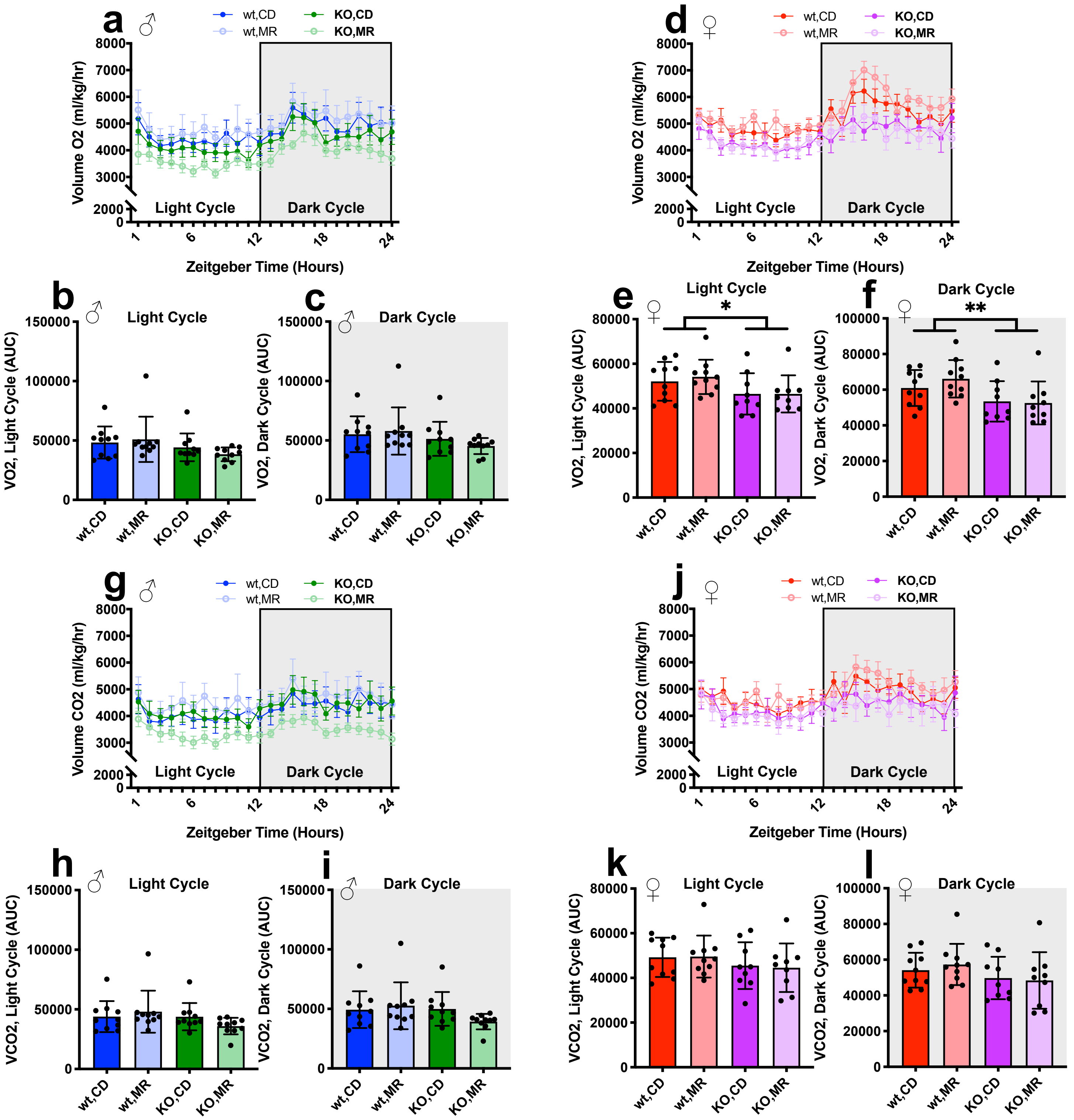

Supplemental Figure S3 – MR and MsrA did not alter lean weight normalized respirometry
Oxygen consumption (VO2) and carbon dioxide production (VCO2) for 24hr period normalized to lean mass, analyzed via 3-Way ANOVA for light and dark cycle independently (**a**,**d**,**g**,**j**). Graphs represent mean ± s.e.m. Male and female light cycle and dark cycle Area Under the Curve (AUC) were analyzed within each sex via Two-Way ANOVA for main effects on AUC for VO2 (**b**,**c**,**e**,**f**) and VCO2 (**h**,**i**,**k**,**l**). Post-hoc analysis was performed with Sidac multiple comparisons correction to assess the diet effect within each genotype. Line graphs represent means ± s.e.m., bar graphs represent means ± s.d., and all groups were 9-10 mice on diet for ~8 months. (*$p < 0.05$; **$p < 0.01$)

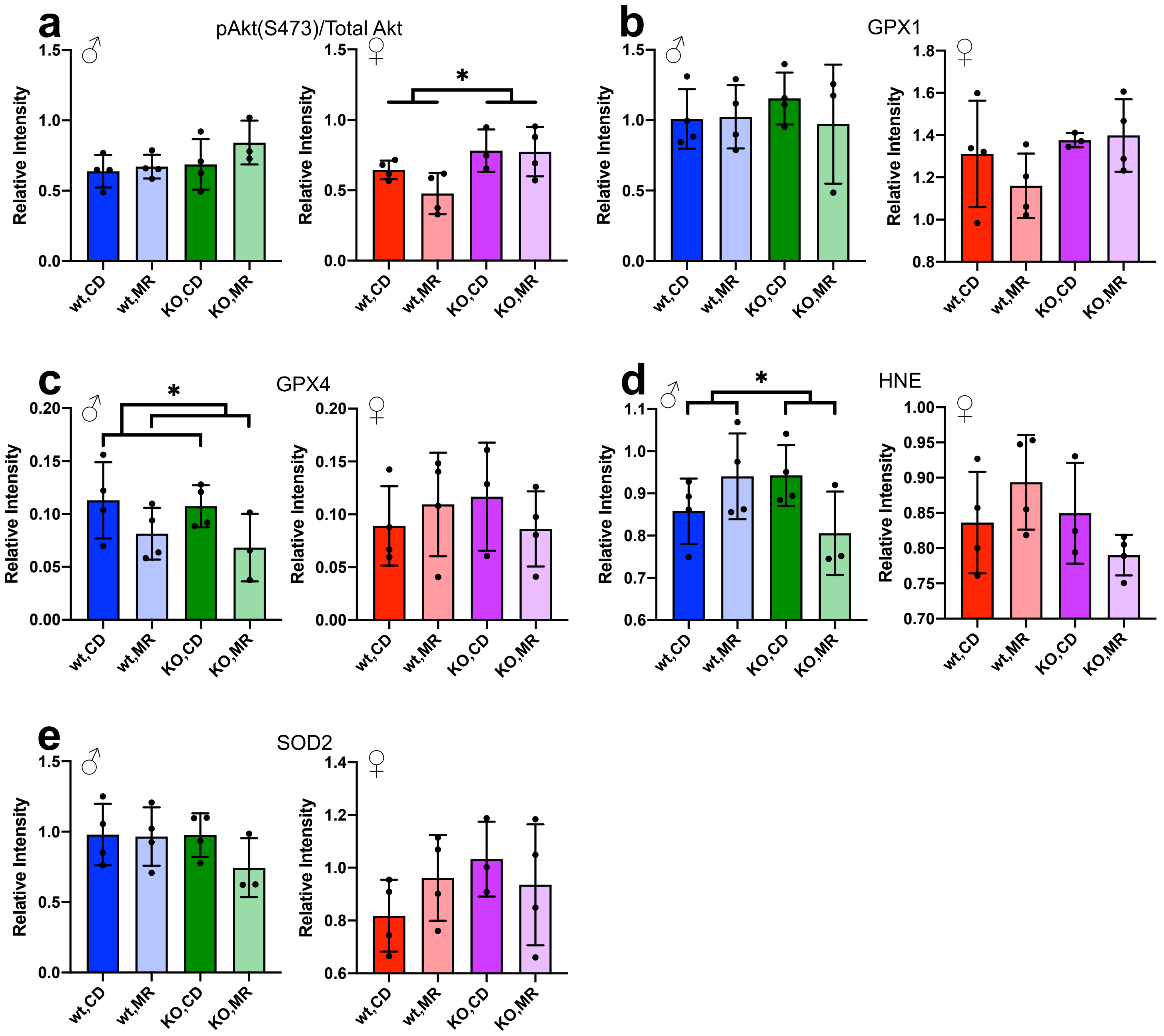

Supplemental Figure S4 – MR and MsrA have minimal effect on liver oxidative stress proteins
Various proteins as measured by Western Blot from liver homogenates – pAkt(S473) to Total Akt ratio (**a**), GPX1 (**b**), GPX4 (**c**), 4-Hydroxynonenal (HNE) (**d**), and SOD2 (**e**). Expression normalized to total protein by Ponceau S staining and to a control sample for between membrane comparison. Analysis was within each sex via Two-Way ANOVA for main effects. Post-hoc analysis was performed with Sidac multiple comparisons correction to assess the diet effect within each genotype. Graphs represent means ± s.d. and all groups were 3-4 mice. (*$p < 0.05$)

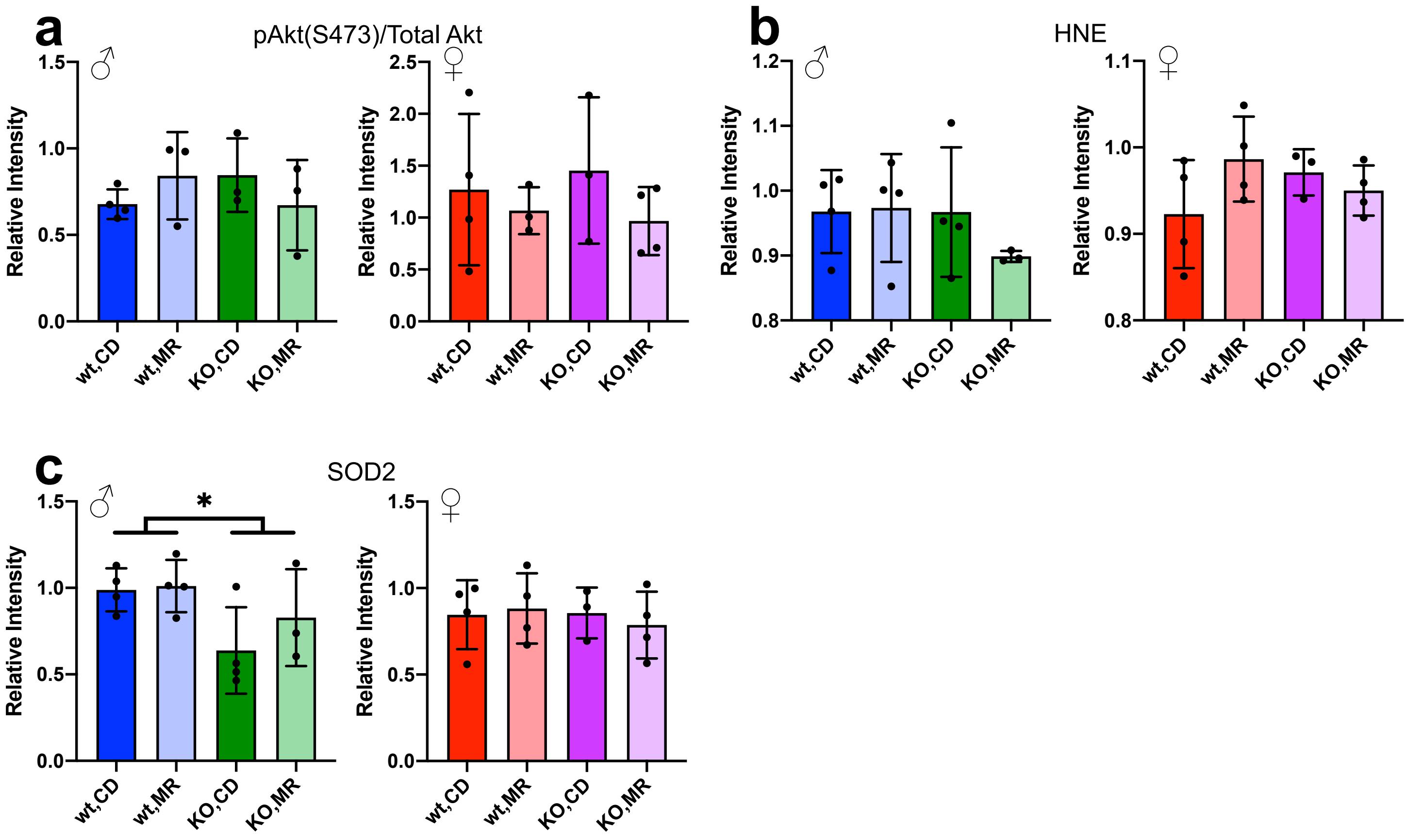

Supplemental Figure S5 – MR and MsrA have minimal effect on skeletal muscle oxidative stress proteins

Various proteins as measured by Western Blot from skeletal muscle homogenates – pAkt(S473) to Total Akt ratio (**a**), 4-Hydroxynonenal (HNE) (**b**), and SOD2 (**c**). Expression normalized to total protein by Ponceau S staining and to a control sample for between membrane comparison. Analysis was within each sex via Two-Way ANOVA for main effects. Post-hoc analysis was performed with Sidac multiple comparisons correction to assess the diet effect within each genotype. Graphs represent means ± s.d. and all groups were 3-4 mice. (*p < 0.05)
